# The Spontaneous Activity Pattern of the Middle Occipital Gyrus Predicts the Clinical Efficacy of Acupuncture Treatment for Migraine Without Aura

**DOI:** 10.3389/fneur.2020.588207

**Published:** 2020-11-09

**Authors:** Tao Yin, Guojuan Sun, Zilei Tian, Mailan Liu, Yujie Gao, Mingkai Dong, Feng Wu, Zhengjie Li, Fanrong Liang, Fang Zeng, Lei Lan

**Affiliations:** ^1^Acupuncture and Tuina School/The 3rd Teaching Hospital, Chengdu University of Traditional Chinese Medicine, Chengdu, China; ^2^Acupuncture and Brain Science Research Center, Chengdu University of Traditional Chinese Medicine, Chengdu, China; ^3^Department of Gynecology, Hospital of Chengdu University of Traditional Chinese Medicine, Chengdu, China; ^4^College of Acupuncture and Moxibustion and Tui-na, Hunan University of Chinese Medicine, Changsha, China; ^5^Traditional Chinese Medicine School, Ningxia Medical University, Yinchuan, China; ^6^Department of Acupuncture and Moxibustion, Xinjin Hospital of Traditional Chinese Medicine, Chengdu, China; ^7^Department of Acupuncture and Moxibustion, Changsha Hospital of Traditional Chinese Medicine, Changsha, China; ^8^Key Laboratory of Sichuan Province for Acupuncture and Chronobiology, Chengdu, China

**Keywords:** migraine without aura, acupuncture, multivariate pattern analysis, machine learning, amplitude of low-frequency fluctuation, efficacy prediction

## Abstract

The purpose of the present study was to explore whether and to what extent the neuroimaging markers could predict the relief of the symptoms of patients with migraine without aura (MWoA) following a 4-week acupuncture treatment period. In study 1, the advanced multivariate pattern analysis was applied to perform a classification analysis between 40 patients with MWoA and 40 healthy subjects (HS) based on the z-transformed amplitude of low-frequency fluctuation (zALFF) maps. In study 2, the meaningful classifying features were selected as predicting features and the support vector regression models were constructed to predict the clinical efficacy of acupuncture in reducing the frequency of migraine attacks and headache intensity in 40 patients with MWoA. In study 3, a region of interest–based comparison between the pre- and post-treatment zALFF maps was conducted in 33 patients with MwoA to assess the changes in predicting features after acupuncture intervention. The zALFF value of the foci in the bilateral middle occipital gyrus, right fusiform gyrus, left insula, and left superior cerebellum could discriminate patients with MWoA from HS with higher than 70% accuracy. The zALFF value of the clusters in the right and left middle occipital gyrus could effectively predict the relief of headache intensity (*R*^2^ = 0.38 ± 0.059, mean squared error = 2.626 ± 0.325) and frequency of migraine attacks (*R*^2^ = 0.284 ± 0.072, mean squared error = 20.535 ± 2.701) after the 4-week acupuncture treatment period. Moreover, the zALFF values of these two clusters were both significantly reduced after treatment. The present study demonstrated the feasibility and validity of applying machine learning technologies and individual cerebral spontaneous activity patterns to predict acupuncture treatment outcomes in patients with MWoA. The data provided a quantitative benchmark for selecting acupuncture for MWoA.

## Introduction

Migraine is a chronic paroxysmal neurological disorder characterized by multiphase attacks of moderate or severe headache and reversible neurological and systemic symptoms (1). As the second most disabling neurological disorder in the world (2), migraine severely affects the quality of life of patients and families (3, 4), causes heavy medication overuse (5), and leads to substantial social and financial burdens (6). Nevertheless, the mechanism regarding its pathophysiology remains unclear and the current treatment for migraine is far from satisfactory (7). As a traditional therapeutic method that stimulates certain points of the body with needles, acupuncture has been applied to treat headaches for a long time in China. Its clinical efficacy in relieving headache intensity and reducing the frequency of migraine attacks has also been verified in several clinical trials (8–10). A recent systematic review suggested that acupuncture could indeed reduce the frequency of migraine attacks, and could be considered as a treatment option for migraine (11). Although acupuncture is effective for migraine, certain differences have been noted with regard to its efficacy across different subjects, suggesting that patients' responses to acupuncture treatment may vary considerably. Identifying migraineurs who may benefit from acupuncture before treatment can improve the efficacy of acupuncture and reduce the waste of medical resources.

Based on the machine learning technologies and neuroimaging markers, a large number of studies have been conducted on efficacy prediction in recent years (12–15). These studies illustrated that the baseline functional magnetic resonance imaging (fMRI) and the structural MRI properties contributed significant information for the prediction of post-intervention symptom relief. A recent machine learning study demonstrated that the pre-treatment resting-state functional connectivity between the medial prefrontal cortex and specific subcortical regions could significantly predict the changes of symptom in patients with chronic low back pain receiving 4-week acupuncture treatment (16). The Multivariate Pattern Analysis (MVPA) study also suggested that the baseline white matter microstructure and gray matter volume could aid the identification of the migraine subjects who were sensitive to the placebo acupuncture stimulation (17, 18). These studies indicated that the baseline neuroimaging properties might be the available markers to predict individual responses to acupuncture. This opened up a new and promising avenue for predicting the efficacy of acupuncture treatment.

Previous studies have found that patients with migraine displayed significant alterations in their cerebral spontaneous activity patterns (19–22), and acupuncture treatment could effectively regulate the disrupted cerebral functional activity of these patients (21). Furthermore, the regional cerebral functional activity and the interregional functional connectivity have been detected as the meaningful features for distinguishing patients with migraine from healthy individuals (23, 24). Therefore, we hypothesized that the baseline cerebral spontaneous activity patterns of migraineurs could be used as a reliable predictor of acupuncture responsiveness after migraine treatment.

We conducted this multilevel study using machine learning approaches and whole-brain z-transformed amplitude of low-frequency fluctuation (zALFF), a widely used indicator reflecting the brain spontaneous activity (25–27). In study 1, we performed an advanced MVPA based on Support Vector Classification (SVC) and the whole-brain zALFF features to discriminate patients with migraine without aura (MWoA) from healthy subjects (HS). In study 2, we employed the Support Vector Regression (SVR) to explore whether and to what extent the meaningful classifying features could predict the relief of symptoms of patients with MWoA after a 4-week acupuncture treatment period. In study 3, we investigated the changes in activity patterns of the meaningful predicting features after acupuncture treatment and examined the associations between the changes in the brain and the improvement of the clinical symptoms.

## Methods

### Participants

Sixty patients with MWoA and 46 well-matched HS were enrolled. A total of 20 patients and 6 HS were excluded for kinds of reasons, whereas 40 patients and 40 HS with eligible baseline MRI data were included in study 1 and study 2. After the treatment period, the 33 patients with complete baseline and secondary MRI data were included in study 3. The details of the dropped-out subjects are shown in Supplementary Figure 1.

All the patients were recruited from the outpatients of the Third Teaching Hospital and the campus of Chengdu University of Traditional Chinese Medicine from June 2011 to November 2013. The potential patients with MWoA were diagnosed by a neurologist according to the 2nd Edition International Classification of Headache Disorders for Migraine Without Aura (28). The detailed inclusion and exclusion criteria for the MWoA patients are provided in Supplementary Material 2.

The right-handed, gender- and age-matched HS were recruited by advertisements at the campus of Chengdu University of Traditional Chinese Medicine. These participants were free from any chronic pain or any other organic or functional disorder. Both patients and HS underwent the comprehensive history taking, physical examination, and laboratory examinations. Individuals with abnormal test results were excluded.

### Outcome Measures

The total observation period for patients with MWoA was 8 weeks, which contained a 4-week baseline period and a 4-week treatment period. All the patients were required to complete the semi-structural migraine diaries both at the baseline period and at the treatment period. The frequency of migraine attacks and headache intensity were selected as the clinical outcomes to reflect the severity of symptoms and measure the clinical efficacy of acupuncture treatments. The frequency of migraine attacks was evaluated with monthly migraine days (MMDs). A migraine day was defined as a calendar day with headache meeting criteria for MWoA. The headache intensity was assessed with the 0–10 Visual Analog Scale (VAS), where 0 indicated no migraine and 10 indicated the most severe migraine. Moreover, the accompanying symptoms during migraine attacks (including photophobia, phonophobia, nausea, and vomiting) were also recorded at the baseline period.

### Interventions

Patients were randomly assigned to receive acupuncture treatment with one of three specific acupoint prescriptions, which all had been proven effective and analogous in relieving migraine in our previous clinical trials (9, 29, 30). The details of these three acupoint prescriptions is intruduced in Supplementary Figure 3. In the treatment period, patients with MWoA received 20 sessions of acupuncture treatment in 4 weeks (five sessions per week). In each session, patients received manual acupuncture treatment with the disposable sterile filiform needles for 30 min with *deqi* sensation. Two licensed acupuncturists with at least 3 years of clinical experience administered all the acupuncture protocols. The detailed acupuncture manipulations could be found in our previous study (31).

### Clinical Data Analysis

The demographic characteristics and clinical outcomes were analyzed via SPSS 20.0 software (SPSS Inc. USA). Between-group comparisons were performed with the two-sample *t*-test or the χ^2^ test. As the pre- and post-treatment VAS and MDDs were repeated measurements, the within-group comparisons were conducted with the linear mixed model. The significance threshold was set to *p* < 0.05 (two-tailed). Because this study contained two outcomes (VAS and MMDs), the significance threshold of the within-group comparisons was adjusted by Bonferroni correction.

### MRI Data Acquisition

MRI data were acquired using a 3.0-T Siemens scanner (Siemens AG, Germany) with an 8-channel phase-array head coil at Huaxi Magnetic Resonance Research Center, West China Hospital of Sichuan University, Chengdu, China. Each subject, with eyes blindfolded and ears plugged, underwent a high-resolution three-dimensional T1-weighted imaging (3D-T1WI) and a resting-state blood oxygenation level–dependent functional MRI (BOLD-fMRI) scan. The 3D-T1WI sequence was acquired with an axial fast spoiled gradient recalled sequence. The parameters were set as follows: repetition time (TR)/echo time (TE) = 1,900/2.26 ms, slice thickness = 1 mm, field of view (FOV) = 256 × 256 mm^2^, and matrix size = 256 × 256. The BOLD-fMRI sequence was obtained with echo-planar imaging. The parameters were set as follows: TR/TE = 2,000/30 ms, flip angle = 90°, slice number = 30, slice thickness = 5 mm, FOV = 240 × 240 mm^2^, matrix size = 64 × 64, and total volumes = 180.

### fMRI Data Preprocessing and zALFF Calculation

The fMRI data preprocessing was carried out with SPM12 (SPM12, http://www.fil.ion.ucl.ac.uk/spm) and DPARSF 4.5 (32) (http://rfmri.org/DPARSF). The main steps included the following: (1) discarding the first 10 timepoints; (2) slice-timing correction, realignment, and discarding subjects with a mean framewise displacement value exceeding 0.5 mm or a maximum displacement greater than one voxel size (33, 34); (3) reorienting functional and T1 images with six rigid-body parameters; (4) coregistering T1 images to functional space, segmentation, and normalizing the functional images to Montreal Neurological Institute (MNI) space; (5) correcting head motion with Friston 24-parameter model (35, 36), removing linear trend, and regressing out the white matter and cerebrospinal fluid signals; (6) re-sampling the functional images to 3-mm^3^ cubic voxels and smoothing functional images with a 6-mm Gaussian kernel of full width at half maximum; (7) temporally filtering (0.01–0.08 Hz) (37) to generate the ALFF maps; (8) transforming the ALFF map to the zALFF map with normal z transformation.

### Study 1: Pattern Classification of Patients With MWoA and HS

The classification analysis between patients with MWoA and HS was performed with the advanced MVPA (38, 39). The input of MVPA was the preprocessed zALFF maps of patients and HS and the output was a 3D spacial map of classification accuracy. The MVPA mainly included the three following steps: feature selection, model construction, and performance evaluation. Considering the curse of dimension caused by high-dimension features of neuroimaging data, an appropriate feature selection approach need to be adopted to filter the redundant features, which could effectively improve the performance of classifiers (40, 41). Initially, the inputted zALFF maps were masked with an Anatomical Automatic Labeling 116 gray matter atlas to remove the signals of white matter and cerebrospinal fluid and reduce the computation burdens. We combined the searchlight algorithm (42) and principal component analysis (PCA) to conduct feature selection referencing the previous studies (43–46). Briefly, at each voxel (Vi), a spherical cluster with a 9-mm radius was defined centering at Vi, and the value of each voxel within the spherical cluster was extracted to generate the feature matrices. Subsequently, PCA was applied to reduce feature dimension and yield the eigenvectors. In the present study, the cumulative contribution threshold of PCA was set to 90%. After feature selection and dimension reduction with PCA, a linear kernel SVC with the default parameters was applied to construct the classification model based on the LIBSVM toolbox (47) (https://www.csie.ntu.edu.tw/~cjlin/Libsvm). Because of the relatively small sample size, the leave-one-out cross-validation (LOOCV) strategy was adopted to evaluate the accuracy of the classifier. In each LOOCV experiment, N – 1 subjects were assigned to the training set, and the remaining one was used as a test sample. The accuracy of each Vi was generated by averaging all accuracy points obtained at each LOOCV test. The aforementioned steps were repeated to calculate the accuracy for all voxels and construct the 3D spatial map of the classification accuracy. The meaningful classifying features were defined as those clusters with a minimum accuracy of 70% and more than 50 contiguous voxels. Same to the previous studies (44, 45), the accuracy of each cluster was determined by its peak accuracy.

In addition, the values of all the voxels containing in the meaningful classifying features were extracted as the input features to build an overall linear kernel SVC for the classification of the 40 MWoA patients and 40 HS. The performance of the classifier was evaluated based on accuracy, specificity, sensitivity, and the area under the curve (AUC). The statistical significance of the classifier performance was measured by permutation testing (permutation times = 5,000).

To evaluate the associations between the classifying features and the severity of symptoms, voxel-based correlation analyses (48) were conducted between the meaningful classifying features and the duration/baseline MMDs/baseline VAS scores in patients with MWoA. The statistical significance threshold of the correlation was set to *p* < 0.05 (uncorrected) and the threshold of cluster size was set to 20 contiguous voxels. The results of the correlation analyses were displayed with the 3D correlation maps. Furthermore, we extracted the average zALFF value of each suprathreshold cluster and plot the correlation scatterplots between these classifying features and the corresponding clinical data.

### Study 2: Prediction of the Efficacy of Acupuncture Treatment From Meaningful Classifying Features

These meaningful classifying features which could effectively distinguish patients with MWoA from HS were used as the feature-of-interest to predict the clinical efficacy of acupuncture in reducing the frequency of migraine attacks (measured with MMDs) and headache intensity (measured with VAS scores). The prediction models were constructed with SVR based on the LIBSVM toolbox. The linear kernel with default parameters and the 10-fold cross-validation were utilized in the prediction models. To assess the performance of prediction models, we calculated the squared prediction–outcome correlation (*R*^2^), which was defined as the squared correlation between the actual and predicted value, as well as the mean squared error (MSE), which was defined as the expectation of the square of the difference between the actual and predicted value. The significance of *R*^2^ and MSE was measured by permutation testing (permutation times = 5,000). To reduce the bias introduced by randomly dividing the subjects during the 10-fold cross-validation, the prediction procedure was repeated 100 times. Finally, the *R*^2^ and MSE were described as mean ± SD of the 100 repetitions.

**Figure 1:**
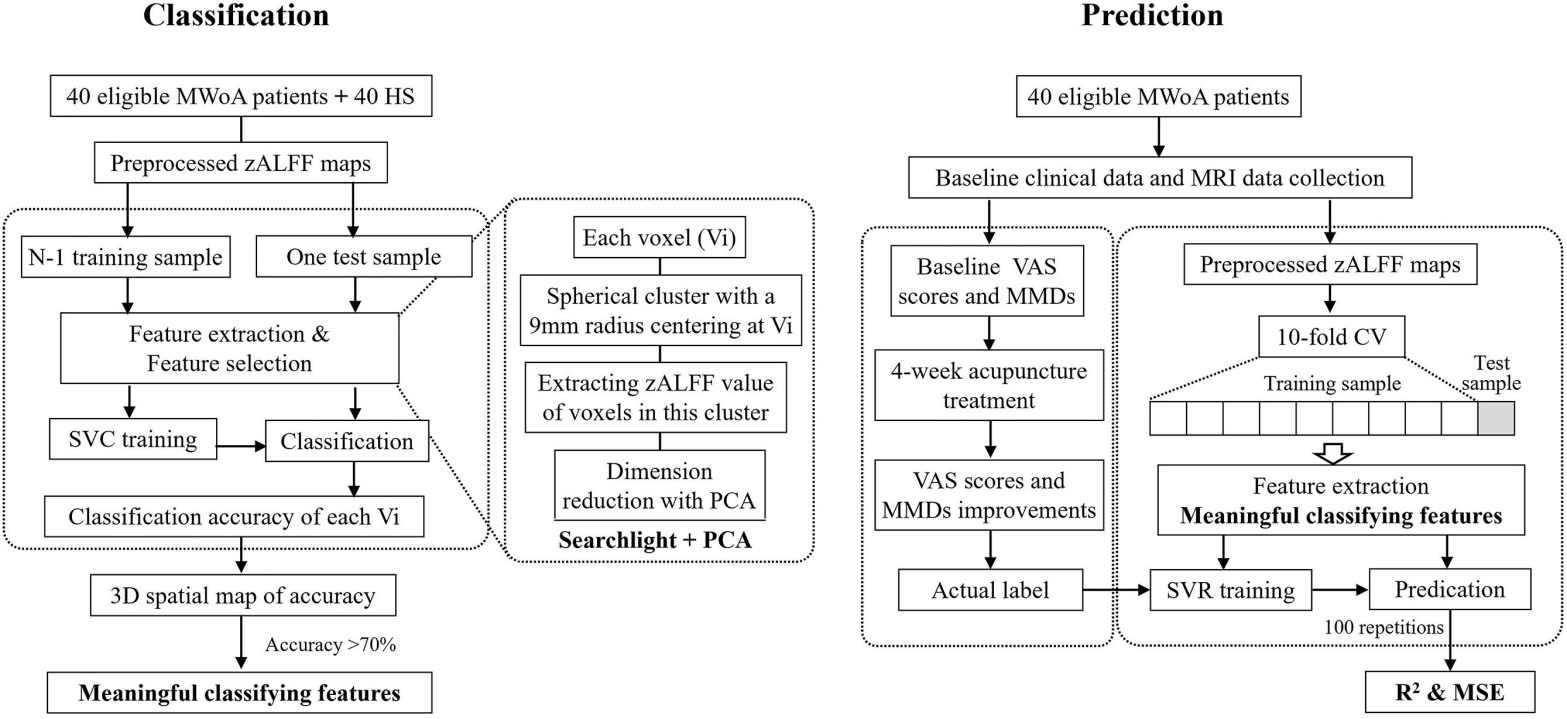
Flow chart of classification and prediction analyses. MWoA, migraine without aura; HS, healthy subjects; zALFF, z-transformed amplitude of low-frequency fluctuation; SVC, Support Vector Classifier; PCA, principal component analysis; MMDs, monthly migraine days; VAS, Visual Analog Scale; CV, cross-validation; SVR, Support Vector Regression; MSE, mean squared error.

The flowchart of the classification and prediction analyses is shown in Figure 1.

### Study 3: Alterations of the zALFF Value in Meaningful Predicting Features After Treatment

To examine the alterations of spontaneous activity of predicting features after acupuncture treatment, the meaningful predicting features identified in the prediction analysis were regarded as regions of interest (ROIs), and an ROI-based comparison was performed between the pre- and post-treatment zALFF maps in patients with MWoA. The significance threshold of the paired *t*-test was set to *p* < 0.05 with family-wise error (FWE) correction. Finally, the average zALFF value of each ROI was extracted to conduct correlation analysis between the improvement of symptoms and the zALFF alterations.

## Results

### Demographic Characteristics and Clinical Outcomes

The characteristics and clinical outcomes of patients with MWoA and HS are summarized in Table 1. After 20 sessions of acupuncture treatment, the patients indicated significant improvement in their VAS scores (*p* = 6 × 10^−5^, *p*_Bonferroni_ < 0.05), whereas reduction in MMDs did not reach statistical significance (*p* = 0.086).

**Table 1:** Demographic characteristics and clinical outcomes of patients with MWoA and HS in studies 1–3.

			**MWoA**	**HS**	***p* value**
Studies 1 and 2	Gender (male/female)		10:30	10:30	1
	Age (years)		21.7 ± 2.15	21.1 ± 2.01	0.201
	Duration (months)		53.75 ± 28.45		
	Baseline VAS scores		5.49 ± 1.14		6 × 10^−5^*^^
	Post-treatment VAS scores		3.80 ± 1.60		
	Baseline MMDs		6.63 ± 6.24		0.086
	Post-treatment MMDs		5.18 ± 5.10		
	Accompanying symptoms (yes/no)	Photophobia	19/21		
		Phonophobia	23/17		
		Nausea	20/20		
		Vomiting	7/33		
Study 3	Gender (male/female)		8:25		
	Age (years)		21.42 ± 1.86		
	Duration (months)		53.12 ± 26.12		
	Baseline VAS scores		5.41 ± 1.12		4 × 10^−5^*^^
	Post-treatment VAS scores		3.68 ± 1.68		
	Baseline MMDs		6.97 ± 6.63		0.078
	Post-treatment MMDs		5.33 ± 5.30		

*MWoA, migraine without aura; HS, healthy subjects; VAS, Visual Analog Scale; MMDs, monthly migraine days*.

* **p* < 0.05, Bonferroni correction*.

### Pattern Classification

**Figure 2:**
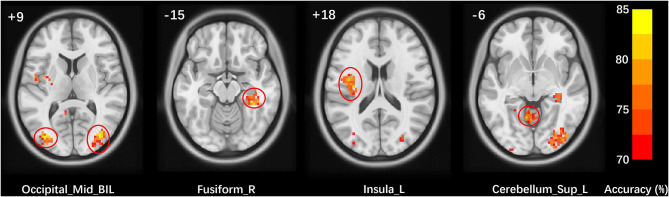
Meaningful classifying features to discriminate MWoA patients and HS. Mid, middle; Sup, superior; BIL, bilateral; R, right; L, left.

As demonstrated in Figure 2 and Table 2, five clusters including the bilateral middle occipital gyrus, right fusiform gyrus, left insula and left superior cerebellum were defined as meaningful classifying features that could discriminate MWoA from HS with higher than 70% accuracy. The voxel with the highest accuracy of 85% was located at the right middle occipital gyrus.

**Table 2:** Meaningful classifying features to discriminate patients with MWoA from HS.

**Region (hemisphere)**	**Cluster size (voxels)**	**MNI coordinates**			**Peak accuracy (%)**
		**X**	**Y**	**Z**	
Middle occipital gyrus (R)	289	36	−75	6	85.00
Middle occipital gyrus (L)	104	−36	−81	9	82.50
Fusiform gyrus (R)	128	33	−36	−15	83.75
Insula (L)	127	−33	−15	18	81.25
Superior cerebellum (L)	118	−9	−57	−6	80.00

*R, right; L left; MNI, Montreal Neurological Institute*.

Using the zALFF values of voxels in these five clusters as inputted features, the overall linear kernel SVC achieved an accuracy of 86.25% (*p* = 0.0002), AUC of 0.9213 (*p* = 0.0002), sensitivity of 87.5%, and specificity of 85%.

**Figure 3:**
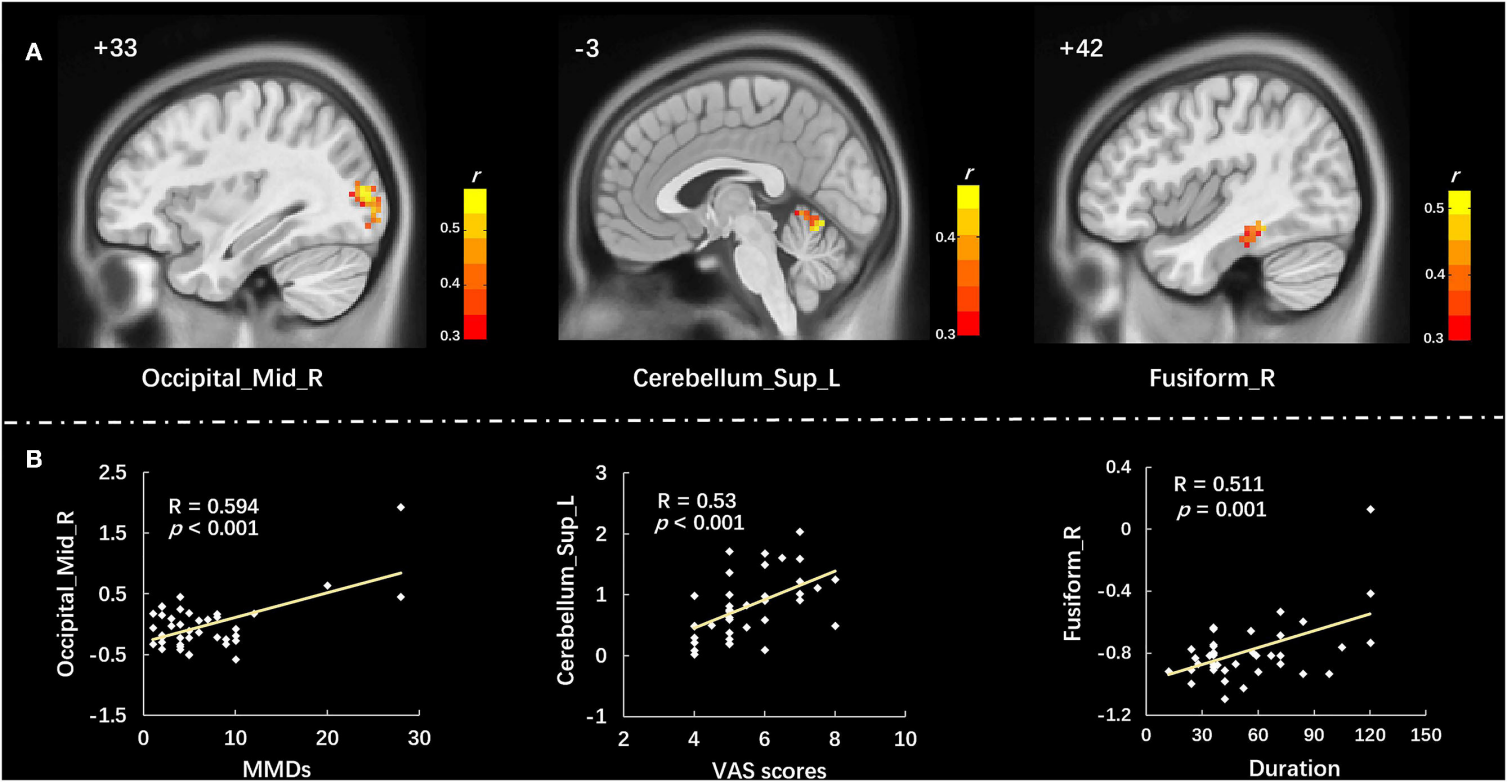
Results of correlation analyses between zALFF value of meaningful classifying features and clinical characteristics. **(A)** Results of voxel-based correlation analyses. **(B)** Correlation scatterplots between the average zALFF value of each suprathreshold cluster and clinical characteristics. Mid, middle; Sup, superior; R, right; L, left; MMDs, monthly migraine days; VAS, Visual Analog Scale.

The results of the voxel-based correlation analysis illustrated that the zALFF value of the right middle occipital gyrus exhibited a positive correlation with baseline MMDs (cluster size = 101, peak *r* = 0.5778), the zALFF value of the left superior cerebellum positively correlated with the baseline VAS scores (cluster size = 40, peak *r* = 0.4535), and the zALFF value of the right fusiform gyrus correlated positively with the duration (cluster size = 21, peak *r* = 0.4911) in patients with MWoA (Figure 3A). Figure 3B displays the correlation scatterplots between the average zALFF value of each suprathreshold cluster and the corresponding baseline clinical characteristics.

### Prediction of Acupuncture Efficacy

**Figure 4:**
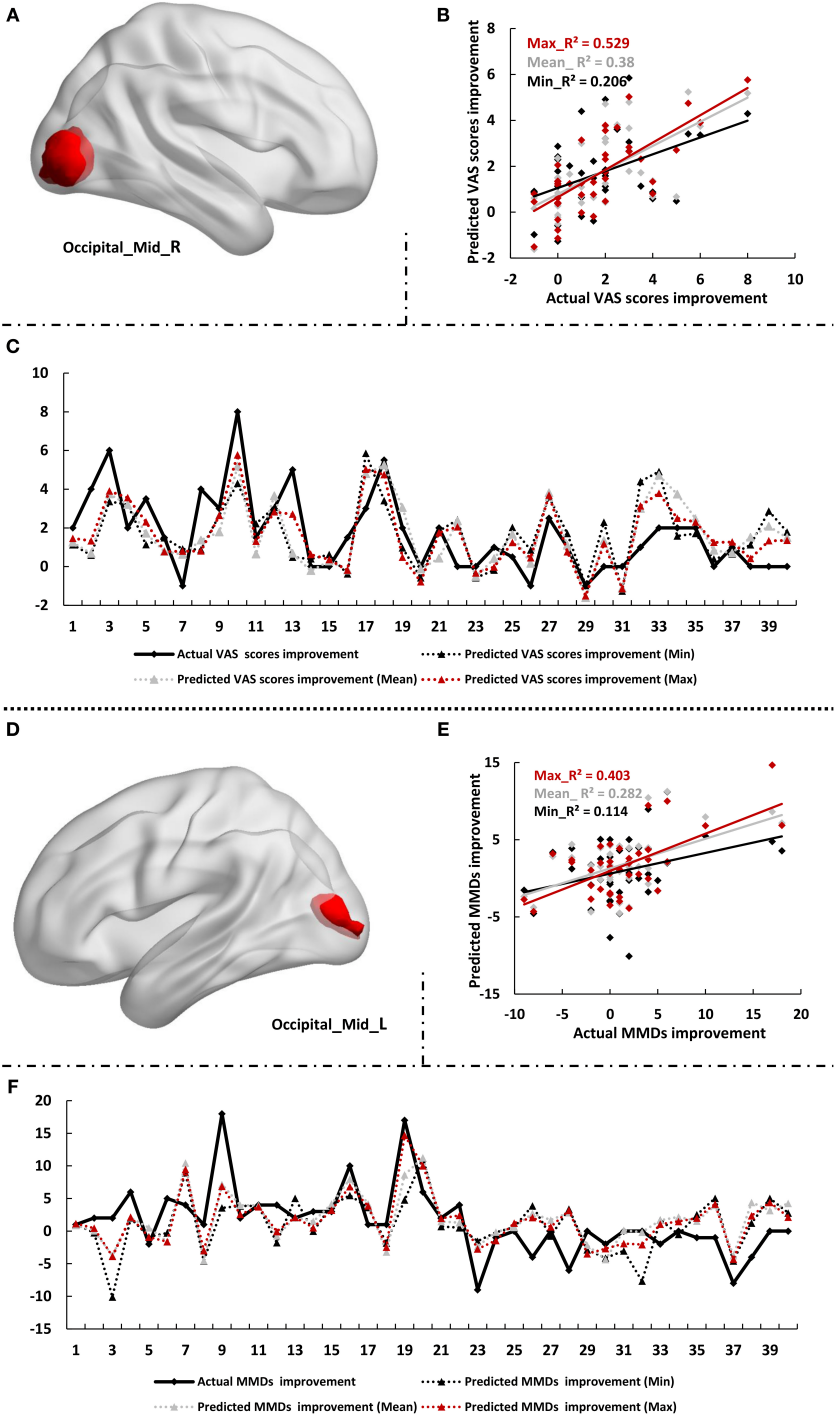
Results of prediction analyses. **(A–C)** Results of prediction for VAS scores improvement. **(D–F)** Results of prediction for MMDs improvement. **(A,D)** were the defined ROIs. **(B,E)** were the correlation scatterplots between the actual values and predicted values, the scatterplots for the best predictive performance (Max), the worst predictive performance (Min), and the average predictive performance (Mean) in the 100 repetitions were plotted, respectively. **(C,F)** were the line chart reflecting coincidence of actual and predicted values at the condition of Max, Min, and Mean. Mid, middle; R, right; L, left; VAS, Visual Analog Scale; MMDs, monthly migraine days.

These five clusters which defined as the meaningful classifying features in MVPA were selected as feature of interest in prediction analyses. We found that clusters of the right and left middle occipital gyrus contributed significantly to the prediction of acupuncture efficacy. The zALFF value of the right middle occipital gyrus provided significant information to predict the improvement of VAS scores after a 4-week acupuncture treatment period (*R*^2^ = 0.38 ± 0.059, *p* = 0.002 ± 0.003; MSE = 2.626 ± 0.325, *p* = 0.001 ± 0.001). Furthermore, the zALFF value of the left middle occipital gyrus could effectively predict the relief of MMDs after a 4-week acupuncture treatment period (*R*^2^ = 0.282 ± 0.072, *p* = 0.014 ± 0.031; MSE = 20.535 ± 2.701, *p* = 0.008 ± 0.014) (Figure 4). Supplementary Table 4 displays a summary of the predication performance using each cluster as an ROI to predict the improvement of VAS scores and MMDs.

### zALFF Alteration After Acupuncture Treatment

**Figure 5:**
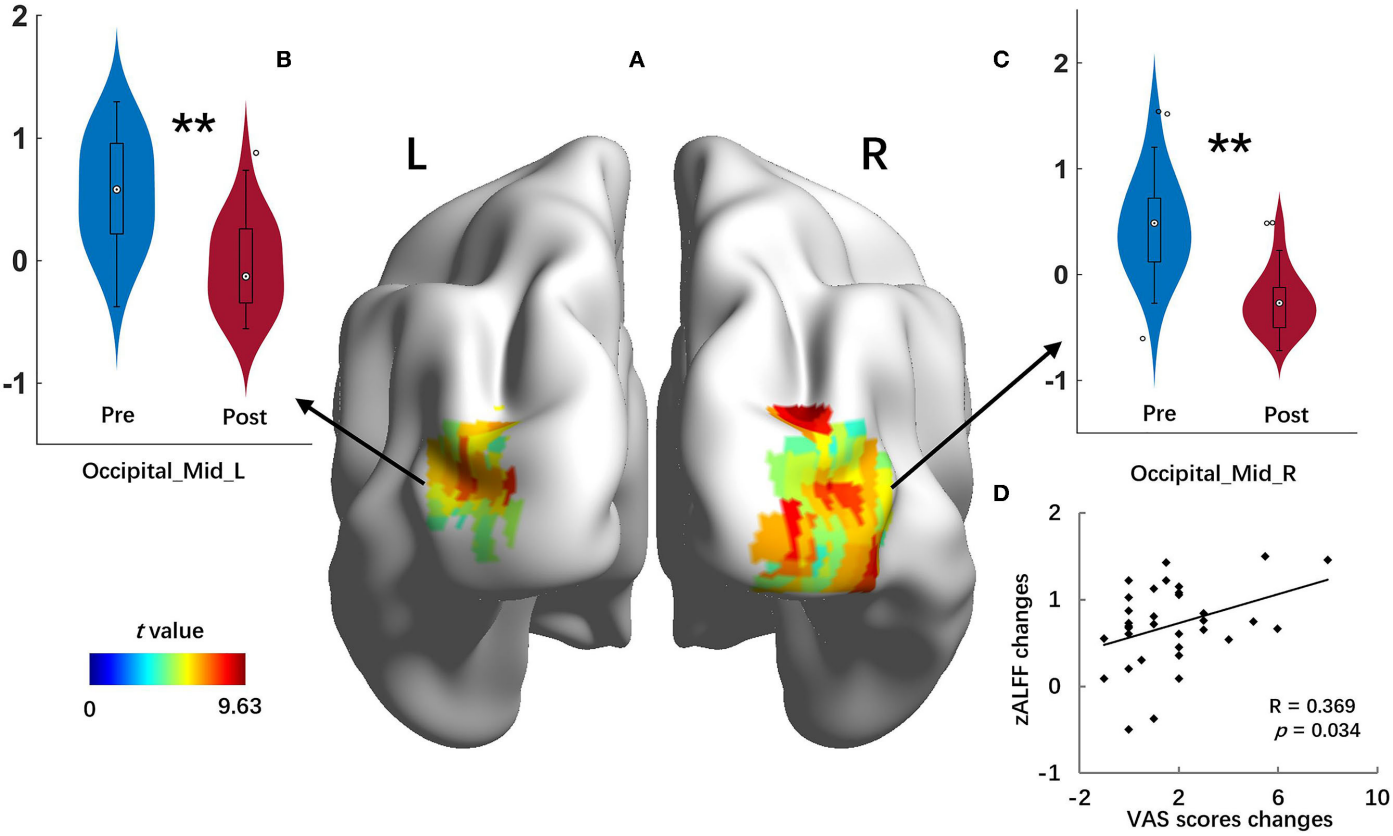
zALFF changes of the right and left middle occipital gyrus after treatment. **(A)** Results of ROI-based comparison between pre- and post-treatment zALFF maps of 33 patients with MWoA; **(B,C)** violin plot of zALFF changes in the left and right middle occipital gyrus after treatment; **(D)** correlation scatterplot between the improvement of VAS scores and zALFF changes in the right middle occipital gyrus. Mid, middle; L, left; R, right; VAS, Visual Analog Scale. ^**^p < 0.01.

The right and left middle occipital gyrus were selected as ROIs. The results of the ROI-based paired *t*-test indicated significant zALFF decrease in the right middle occipital gyrus [voxel size = 215, MNI coordinate (X Y Z) = 30 −75 15, *t* = 9.63] and the left middle occipital gyrus [voxel size = 65, MNI coordinate (X Y Z) = −30 −81 12, *t* = 8.31] after acupuncture treatment (*p* < 0.05, FWE corrected). The changes of the zALFF value in the right middle occipital gyrus exhibited a significant positive correlation with the VAS score improvement (Figure 5).

## Discussion

This was the first study to predict the clinical efficacy of acupuncture for migraine using functional neuroimaging markers. The results demonstrated the feasibility of predicting acupuncture efficacy with machine learning technologies and individual distinct cerebral spontaneous activity patterns. In this multilevel study, we first perform a classification analysis between patients with MWoA and HS and demonstrated that the zALFF value of the foci in bilateral middle occipital gyrus, right fusiform gyrus, left insula, and left superior cerebellum could discriminate patients with MWoA from HS with higher than 70% accuracy. Treating these meaningful classifying features as feature of interest, we constructed the SVR models to predict the acupuncture efficacy in patients with MWoA and detected that the zALFF value of the right and left middle occipital gyrus could effectively predict the relief of headache intensity and frequency of migraine attacks, respectively. In addition, after the 4-week acupuncture treatment, the zALFF value of the predicting features was significantly decreased and this alteration was positively correlated with the relief of headache intensity.

The present study illustrated that the spontaneous activity pattern of the bilateral middle occipital gyrus not only reflected the pathophysiology of MWoA but also exhibited the potential to predict the responsiveness of acupuncture treatment. Our recent machine learning study (49) demonstrated that the decreased resting-state functional connectivity within the middle occipital gyrus could serve as a connectome marker for classifying patients with MWoA and HS. Taken current findings together, we suggested that alterations of the activity pattern in middle occipital gyrus were the features that contributed significantly to the identification of MWoA cases.

The occipital cortex is the visual perception and processing center. Given the complex functional connection of the temporo-occipital cortex, the occipital cortex is also responsible for multi-sensory integration of visual, auditory, and tactile information (50–52). Various studies have previously illustrated structural and functional alterations of the occipital cortex in patients with migraine (22, 53–55), which are generally thought to be associated with the aura phenomenon, especially visual aura (56). Interestingly, these significant structural and functional alterations of the occipital cortex were also observed in patients with MWoA (57–62). For example, in our recent study (61), a strong positive dynamic connectivity within the visual cortex and the significantly negative dynamic connectivity between middle occipital gyrus and posterior thalamus were found in MWoA patients. Moreover, the connectivity between middle occipital gyrus and posterior thalamus correlated significantly with migraine frequency. A structural MRI study (59) further demonstrated that the gray matter volume of the middle occipital gyrus exhibited significant associations with migraine frequency and migraine duration of the patients with MWoA, which was consistent with our findings to some extent. These results implied that the structural and functional abnormalities of the occipital cortex, notably of the middle occipital cortex, were not only associated with the specific changes of migraine with aura but were also considered an important pathological feature of MWoA. The hyperexcitability of the occipital cortex was considered the neural basis of the photophobia or hypersensitivity to light (63, 64). In the present study, more than 75% (31/40) of MWoA patients experienced photophobia and/or phonophobia during migraine attacks. Therefore, we speculated that the recurrent onsets of these symptoms during migraine attacks were associated with the disturbed integration of visual and auditory signals caused by the abnormal spontaneous activity of the occipital cortex. The significant correlation between baseline MMDs and the zALFF value of the right middle occipital gyrus supported this speculation.

Our previous study (65) demonstrated glucose metabolism changes in the middle occipital cortex of migraineurs who were stimulated with electroacupuncture. In a recent study (49), we further detected the significant correlations between the changes of functional connectivity pattern in the occipital cortex and the improvement of migraine frequency after acupuncture treatment. These findings suggested that the occipital cortex might be a potential target of acupuncture treatment for migraine. Interestingly, our current results implicated the feasibility of predicting the clinical efficacy of acupuncture treatment for MWoA using the spontaneous activity patterns of middle occipital gyrus. Moreover, the significant changes in cerebral activity of the bilateral middle occipital gyrus were also directly observed after acupuncture treatment. These findings further expanded our understanding regarding the important role of the occipital cortex in acupuncture treatment for migraine. Considering the associations between the abnormal functional activity of the middle occipital gyrus and the migraine symptoms, as well as the significant changes in functional activity of the middle occipital gyrus after acupuncture treatment, we believed that the spontaneous activity pattern of the middle occipital gyrus predicting the efficacy of acupuncture was not a coincidence.

In addition to the bilateral middle occipital gyrus, the spontaneous activity patterns of the foci in the right fusiform gyrus, left insula, and left superior cerebellum were also deemed meaningful for the discrimination of the patients with MWoA and HS. The fusiform gyrus is a component of the visual recognition network and participates in high-level visual processing, notably in object recognition and category identification (66). As mentioned before, significant alterations in the visual cortex were also noted for migraineurs without visual aura. As an auxiliary region of visual processing, the spontaneous activity pattern in the fusiform gyrus of patients with MWoA was identified as different from that of HS in the present study. The fusiform gyrus has also been shown to be associated with pain perception and cognitive processing (67). This may explain the detection of the disrupted functional activity in the fusiform gyrus in a variety of pain disorders, including migraine (20, 68), fibromyalgia (67), and chronic low back pain (69).

Migraine is a disorder with the global dysfunction of multisensory integration, cognition and attention, affective processing, and executive function (70). As a core node of ascending pain pathways and descending pain modulatory pathways (71), the insula is regarded as the cortical hub of migraine (72). Functional and structural alterations of the insula would specifically and extensively induce the representation of migraine symptoms, including pain, autonomic dysfunction, and cognition errors. Previous studies have illustrated the abnormal functional activity and functional connectivity pattern of the insula in migraineurs from the group level (20, 73). In the present study, we further demonstrated the individual differences in the spontaneous activity patterns of the insula between patients with MWoA and HS.

As an important region closely associated with the pathophysiology of migraine, the cerebellum is proposed to be linked to the sensorimotor functions and cognitive and pain information processing (74, 75). It has been shown that the cerebellum is activated during trigeminal nociceptive stimulation and that the magnitudes of its activity are modulated by the perceived intensity of pain (76). A significant correlation between migraine frequency and BOLD signals of the cerebellum was also shown in migraineurs under the trigeminal nociceptive stimulation (77). These results demonstrated that the activity of the cerebellum was strongly associated with pain attack induced by the nociceptive stimulation. Furthermore, the cerebellum involvement was observed in MWoA, migraine with aura, basilar-type migraine, and familial hemiplegic migraine (78), which all indicated that the cerebellum participated in the pathophysiological mechanism of migraine extensively. Based on these findings, we concluded that the alterations of spontaneous activity patterns of the fusiform gyrus, insula, and cerebellum which involved pain perception, pain processing, and multisensory integration were the important central pathological features that distinguished patients with MWoA from HS.

There are several limitations in the current study. First, this study lacks a sham acupuncture intervention, so the placebo effects of acupuncture could not be ruled out. Notably, the primary purpose of the current study was to explore whether and to what extent the neuroimaging markers could predict clinical efficacy of acupuncture for MWoA patients. The similarities and differences in neuroimaging features that predict the clinical efficacy of real or sham acupuncture intervention could be the focus of future researches. Second, all the MRI data were derived from the same center. It is required to validate the results in fully independent samples from multi-centers to ensure the robustness of the classifiers and predictors. Third, the sample size was relatively small. According to Chen et al.'s study (79), a sample size of 40 or higher should be used per group to ensure the test–retest reliability and sensitivity of the results in fMRI studies. In the present study, we performed MVPA based on 40 patients with MWoA and 40 HS. Therefore, the findings were considered reliable and stable. Fourth, the treatment lasted only 4 weeks and the findings only indicated the short- to mid-term effects of acupuncture. Our recent study has shown the long-term efficacy of acupuncture in patients with MWoA (9). Future studies should focus on the prediction of the long-term efficacy of acupuncture treatment.

## Conclusion

In conclusion, the present study identified the spontaneous activity pattern of bilateral middle occipital gyrus, right fusiform gyrus, left insula, and left superior cerebellum as potential classifying features for distinguishing patients with MWoA and predicted the symptom relief after acupuncture treatment using the spontaneous activity pattern of the bilateral middle occipital gyrus. This study demonstrated the feasibility and validity of applying machine learning technologies and individual functional cerebral measurements to predict acupuncture treatment outcomes in patients with MWoA. The present findings provided a quantitative benchmark for selecting acupuncture for migraine and may aid the screening of patients who respond well to acupuncture and optimize the allocation of medical resources.

## Data Availability Statement

All datasets generated for this study are included in the article/Supplementary Material.

## Ethics Statement

The studies involving human participants were reviewed and approved by the Ethics Committee of Hospital of Chengdu University of Traditional Chinese Medicine. The patients/participants provided their written informed consent to participate in this study. Study 3 was registered at ClinicalTrials.gov (NCT01152632). The implementation of this study followed the principles of the Declaration of Helsinki.

## Author Contributions

LL, FZ, and FL conceived and designed the study. GS, ML, YG, MD, and FW recruited the subjects. TY, ZT, and ZL analyzed the data. TY drafted the article. FZ revised the article. All authors contributed to the article and approved the submitted version.

## Conflict of Interest

The authors declare that the research was conducted in the absence of any commercial or financial relationships that could be construed as a potential conflict of interest.
