# Supplementary material for: The Spontaneous Activity Pattern of the Middle Occipital Gyrus Predicts the Clinical Efficacy of Acupuncture Treatment for Migraine Without Aura
Source: Front Neurol. 2020 Nov 9;11:588207. doi: 10.3389/fneur.2020.588207 (PMC7680874; doi:10.3389/fneur.2020.588207)
Supplement: Supplementary file 1 [file Presentation_1.PDF]

Supplementary Material

S1. The details of the dropped-out subjects.

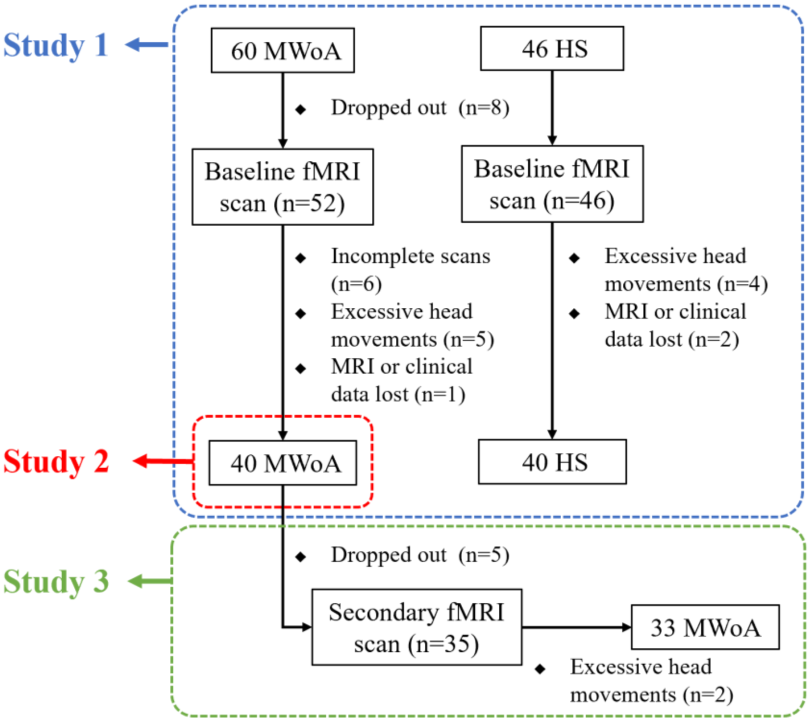

A total of 60 patients with MWoA and 46 well-matched healthy subjects (HS) were enrolled in this study. Of these 106 subjects, 8 patients quitted in the baseline phase and did not participate in the first MRI scan, 6 patients were excluded due to incomplete baseline scans (lack of fMRI or 3DT1 images), 5 patients and 4 HS were excluded due to excessive head movements, 1 patient and 2 HS were excluded due to MRI or clinical data lost. The 40 patients and 40 HS with eligible baseline MRI data were included in study 1, and these 40 patients included in study 2. Five patients dropped out during treatment and did not participate in the secondary MRI scan, and 2 MWoA patients were excluded for the excessive head movements in the secondary MRI scan. Therefore, 33 patients with complete baseline and second MRI data were included in study 3.

S2. The inclusion and exclusion criteria for MWoA patients.

Patients were enrolled if they fulfilled the following inclusion criteria: 1) were 17 to 45 years old, 2) were right-handed, 3) had migraine symptoms for at least six months, 4) had at least one attack per month during the last three months, 5) were absent from taking any analgesics or acupuncture treatment for migraine during the last three months, 6) were moderate or severe headache according to Visual Analogue Scale (VAS)[1] scores. Patients were excluded if they: 1) were secondary headache caused by brain trauma, hypertension, or any other organic reasons, 2) suffered from any psychiatric or neurological diseases or had any severe primary illnesses, 3) were in pregnancy or during lactation, or intended to pregnant in the following six months, 4) suffered from any other chronic pain conditions, 5) suffered from serious anxiety and depression assessed by Self-rating Anxiety Scale (SAS)[2] and Self-Rating depressive Scale (SDS)[3], 6) had any contraindication of MRI scanning or acupuncture.

Reference

[1] Ohnhaus EE, Adler R. Methodological problems in the measurement of pain: a comparison between the verbal rating scale and the visual analogue scale. Pain. 1975;1(4):379-84.

[2] Zung WW. A rating instrument for anxiety disorders. Psychosomatics. 1971;12(6):371-9.

[3] Zung WW. A SELF-RATING DEPRESSION SCALE. Arch Gen Psychiatry. 1965;12:63-70.

S3. Location of acupoints of these three acupoint prescriptions.

Acupoint prescription 1 (A1)

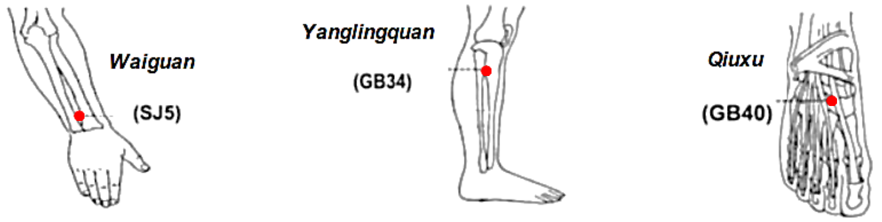

Acupoint prescription 1 (A2)

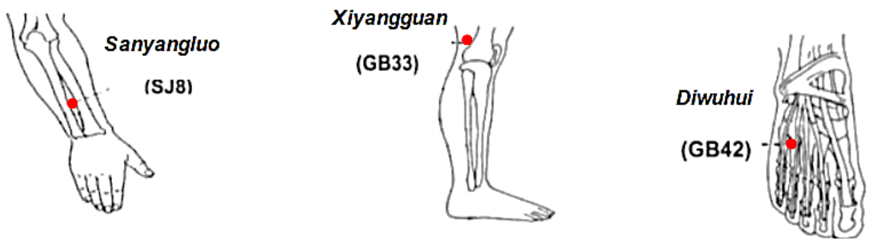

Acupoint prescription 1 (A3)

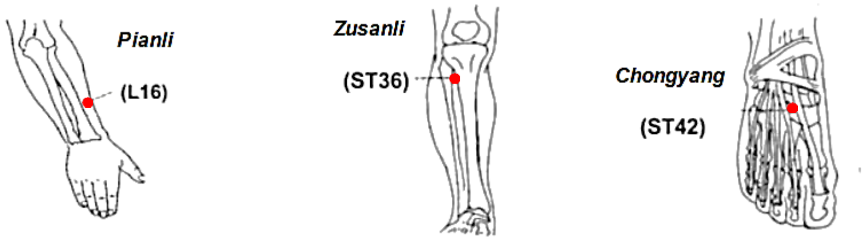

Prescription 1 including bilateral Waiguan (SJ5),Yanglingquan (GB34), Qiuxu (GB40).

Prescription 2 including bilateral Sanyangluo (SJ8), Xiyangguan (GB33), Diwuhui (GB42).

Prescription 3 including bilateral Pianli (L16), Zusanli (ST36) and Chongyang (ST42).

S4. Summary of the predication performance using each meaningful classifying feature as ROI.

ROI (Hemisphere)	Predict improvement of MMDs			
	R^2	MSE	p_R^2	p_MSE
Middle occipital gyrus (R)	0.014±0.016	45.114±5.593	0.654±0.217	0.296±0.126
Middle occipital gyrus (L)	0.284±0.072	20.535±2.701	**0.014±0.031***	**0.008±0.014****
Fusiform gyrus (R)	0.021±0.016	34.656±1.891	0.529±0.169	0.745±0.128
Insula (L)	0.014±0.01	32.76±1.193	0.612±0.156	0.608±0.104
Superior cerebellum (L)	0.127±0.043	25.784±2.154	0.098±0.059	0.029±0.018
All clusters	0.051±0.035	30.816±3.414	0.35±0.221	0.11±0.074

ROI (Hemisphere)	Predict improvement of VAS scores			
	R^2	MSE	p_R^2	p_MSE
Middle occipital gyrus (R)	0.38±0.059	2.626±0.325	**0.002±0.003****	**0.001±0.001****
Middle occipital gyrus (L)	0.074±0.036	11.21±1.103	0.228±0.12	0.779±0.089
Fusiform gyrus (R)	0.007±0.01	5.555±0.443	0.763±0.171	0.366±0.125
Insula (L)	0.031±0.024	5.294±0.576	0.47±0.183	0.3±0.152
Superior cerebellum (L)	0.015±0.014	11.941±1.139	0.621±0.176	0.756±0.097
All clusters	0.011±0.01	5.791±0.483	0.68±0.172	0.322±0.108

ROI, region-of-interest; R, right; L, left; VAS, Visual Analogue Scale; MMDs, monthly migraine days; MSE, mean squared error; *, p<0.05, **, p<0.01.
